# Relationship between soy and isoflavone intake and periodontal disease: The Freshmen in Dietetic Courses Study II

**DOI:** 10.1186/1471-2458-8-39

**Published:** 2008-01-29

**Authors:** Keiko Tanaka, Satoshi Sasaki, Kentaro Murakami, Hitomi Okubo, Yoshiko Takahashi, Yoshihiro Miyake, for the Freshmen in Dietetic Courses Study II Group

**Affiliations:** 1 Department of Public Health, Faculty of Medicine, Fukuoka University, Fukuoka, Japan; 2 Department of Social and Preventive Epidemiology, School of Public Health, The University of Tokyo, Tokyo, Japan; 3 Department of Nutrition Sciences, Kagawa Nutrition University, Saitama, Japan; 4 Department of Health and Nutrition, Wayo Women's University, Chiba, Japan

## Abstract

**Background:**

Much research has shown that soy products inhibited various diseases. However, no published studies have examined the effects of consumption of soy and isoflavones on periodontal disease. The aim of this study was to investigate whether soy and isoflavone intake is associated with the prevalence of periodontal disease.

**Methods:**

The subjects were 3956 Japanese female students, aged 18 to 22 years, who were taking a dietetic course. Periodontal disease was defined as present when a subject reported diagnosis of the disorder by a dentist. Information on dietary factors was collected using a validated diet history questionnaire. Logistic regression analysis was used to estimate the odds ratios and their confidence intervals of periodontal disease. Adjustment was made for cigarette smoking, toothbrushing frequency, region of residence, and body mass index.

**Results:**

The prevalence of periodontal disease was 8.0%. Intake of total soy product and tofu was independently associated with a decreased prevalence of periodontal disease; multivariate odds ratios in comparison of the highest with the lowest quintile were 0.68 and 0.68, respectively (95% confidence intervals = 0.47–0.97 and 0.47–0.98, *P *for trend = 0.01 and 0.004, respectively). A significant inverse dose-response relationship between the intake of isoflavones and the prevalence of periodontal disease was observed, although the difference in the adjusted odds ratio between the extreme quintiles was of borderline significance (*P *for trend = 0.04). There were no measurable dose-response relationships between consumption of tofu products, fermented soybeans, boiled soybeans, miso, or miso soup and the prevalence of periodontal disease.

**Conclusion:**

Our findings suggest that soy and isoflavone intake may decrease the likelihood of periodontal disease. Further investigations with objective measures for periodontal disease are needed to confirm our findings.

## Background

A high level of consumption of soy is intrinsic to the Japanese diet. Soy is a rich dietary source of a group of phytochemicals called isoflavones. Isoflavones have a close similarity in structure to estrogens and are known as phytoestrogens [1-3]. The most abundant active components of isoflavones are genistein and daidzein. Isoflavones are thought to exert a myriad of biological effects and it has been hypothesized that they inhibit various diseases, including hormone-related cancers, cardiovascular disease, osteoporosis, inflammatory diseases, and allergic diseases [1-13]. These effects might be mediated by estrogenic, antiestrogenic and antioxidant activities or other properties of isoflavones [1-3]. Estrogens have been shown to stimulate the immune system [14]. Although the exact role of phytoestrogen on immune activity in humans remains unclear, extensive animal studies showing the effect of isoflavones on immune parameters suggested the feasibility of genistein and daizein as having immunologic effects in humans [15]. An interventional study showed that consumption of high levels of isoflavone modulated cytokine production in women [16]. Thus, it is likely that dietary isoflavone intake would affect the host's immune system.

Periodontal disease is usually a painless, slowly progressive chronic infectious condition that can result in the inflammatory destruction of tooth-supporting tissues such as gingiva, periodontal ligament, and alveolar bone. The balance between host resistance and virulence of the infective process determines the extent of periodontal disease [17].

Clinically, estrogen-sufficient patients are likely to have much periodontal plaque without increased gingival inflammation [18]. Taichman & Eklund showed that current high-dose oral contraceptive use was statistically significantly associated with a decreased prevalence of periodontal disease among non-pregnant, premenopausal females aged 17 to 50 years using cross-sectional data from the first National Health and Nutrition Examination Survey, whereas there was no association between low-dose oral contraceptive use and periodontitis in the third National Health and Nutrition Examination Survey [19]. A cross-sectional study among young Irish women showed that current birth control pill users had deeper probing depth and attachment loss than non-users [20]. Thus, evidence supporting or refuting the association between estrogen levels and periodontal diseases is not yet firmly established.

To our knowledge, no published epidemiological studies have examined the association of soy or isoflavone intake with periodontal disease. We hypothesized that phytoestrogen intake might protect against periodontal disease through a possible effects on the immune system. In the present cross-sectional study, we investigated whether soy and isoflavone intake is associated with the prevalence of periodontal disease among Japanese female young adults. It is worthwhile to study this relationship in Japan where people consume large quantities of soy foods.

## Methods

### Subjects

Study subjects were students who were enrolled in dietetics courses of 54 universities, colleges, and technical schools in 33 of the 47 prefectures in Japan as of April 2005. The background and general procedure of the Freshmen in Dietetic Courses Study II have been described previously [21]. Within 2 weeks after the courses began, a set of 2 self-administered questionnaires on dietary habits and lifestyle over the previous one month was distributed by staff at each school to all 4679 students. Then within 4 weeks of the beginning of the course participants completed a third questionnaire that included questions on lifestyle and health during the previous 6 years. The staff at each school checked the returned questionnaires according to the survey protocol. When missing answers and/or illogical data were detected, the subjects were given another opportunity to answer those questions. A total of 4286 (4066 women and 220 men) students completed the questionnaires (response rate = 91.6%). Because the number of male students was small, as was the number of students over the age of 22 years, the current study was restricted to women aged 18 to 22 years who provided information on variables used in the current study, leaving data on 3956 women available for analysis. The staff at each school explained to students the content of the survey and that participation was voluntary. Written consent was obtained, and this study was approved by the ethical committee of the National Institute of Health and Nutrition.

### Measurements

One of the questionnaires in the set of two was a validated self-administered diet history questionnaire that was used to estimate dietary habits over a period of 1 month. Details of the structure and validity of the questionnaire have been described [22,23]. In this instrument, intake of 147 food items was calculated using an ad hoc computer algorithm developed to analyze the questionnaire. The questionnaire included 6 food items representing soy products (tofu, tofu products such as deep-fried tofu and fried bean curd, fermented soybeans, boiled soybeans, miso, and miso soup). Total soy product intake was considered as the sum of intake of these 6 food items. Soy protein intake was also calculated as the sum of the protein in these food items. Intake of isoflavone (sum of daidzein and genistein intake) was estimated using the food composition table for daidzein and genistein developed especially for Japanese foods [24]. According to validation tests, the energy-adjusted correlation coefficients between the non-consecutive 7-day weighed diet record and the diet history questionnaire were 0.49, 0.48, and 0.49 for isoflavone, daidzein, and genistein, respectively, in another Japanese population (unpublished data). We adjusted for energy intake by the residual method [25]. Our diet history questionnaire also included questions about age, sex, height and weight. Body mass index was calculated by dividing body weight (kg) by the square of height (m).

The second of the self-administered questionnaires elicited information on lifestyle including smoking habits. The third questionnaire requested information on health status such as periodontal status and toothbrushing habits. Periodontal disease was defined as present via a positive answer to the question, "Have you been diagnosed as having periodontal disease by a dentist?".

### Statistical analysis

Intake of selected foods and nutrients was categorized at quintile points on the basis of the distribution of the study population. Cigarette smoking was classified into 2 categories (never, and ever); toothbrushing frequency into 3 (1 or less, 2, and 3+ times/day); and region of residence into 6 (Hokkaido and Tohoku, Kanto, Hokuriku and Tokai, Kinki, Chugoku and Shikoku, and Kyushu). Body mass index was used as a continuous variable.

The present analysis was restricted to female students aged 18 to 22 years. Therefore, sex and age were omitted in the analysis. Covariates included in the multivariate models were cigarette smoking, toothbrushing frequency, region of residence, and body mass index. Two-way interaction between intake of soy or isoflavone and each of the covariates were also examined.

Logistic regression analysis was used to compare the prevalence of periodontal disease with soy and isoflavone intake. Multiple logistic regression analysis was used to control for the potential confounding effects of the selected factors. The trend of association was assessed by a logistic regression model assigning ordinal scores to the levels of the independent variable. Two-sided *P *values less than 0.05 were considered statistically significant. All computations were performed by using the SAS statistical software version 9.1 (SAS Institute, Inc, Cary, NC).

## Results

The prevalence value for periodontal disease was 8.0% among 3956 women. The mean age was 18.1 years (Table 1). The majority of our subjects (97%) were not tobacco users. About 90% of subjects reported toothbrushing twice or more per day. The mean daily total energy and energy-adjusted total soy product consumption were 7709.0 kJ and 45.0 g, respectively (Table 2).

**Table 1:** Distribution of selected characteristics of 3956 Japanese females

Variable	Number (%)
Age (y)	18.1 (0.4)
Cigarette smoking	
Never	3828 (96.8)
Ever	128 (3.2)
Toothbrushing frequency (times/day)	
1 or less	484 (12.2)
2	2899 (73.3)
3+	573 (14.5)
Region of residence	
Hokkaido and Tohoku	332 (8.4)
Kanto	1322 (33.4)
Hokuriku and Tokai	637 (16.1)
Kinki	837 (21.2)
Chugoku and Shikoku	452 (11.4)
Kyushu	376 (9.5)
Body Mass Index (kg/m^2^)	
< 18.5	582 (14.7)
18.5–24.9	3078 (77.8)
25.0–29.9	244 (6.2)
30+	52 (1.3)

**Table 2:** Distribution of daily soy and isoflavone intake in 3956 Japanese females^1^

Variable	Mean (SD)
Total energy (kJ)	7709.0 (2320.4)
Total soy product (g)	45.0 (33.8)
Tofu (g)	20.8 (22.0)
Tofu products (g)	3.0 (5.8)
Fermented soybeans (g)	10.2 (13.3)
Boiled soybeans (g)	4.1 (9.4)
Miso (g)	1.7 (5.3)
Miso soup (g)	5.2 (4.6)
Soy protein (g)	4.6 (3.5)
Isoflavone (mg)	26.8 (21.7)
Daidzein (mg)	10.1 (8.2)
Genistein (mg)	16.7 (13.5)

^1^Nutrition and food intake was adjusted for total energy intake using the residual method.

Odds ratios (ORs) and their 95% confidence intervals (CIs) for relationships between consumption of soy products and the prevalence of periodontal disease are presented in Table 3. Compared with intake of total soy products in the lowest quintile, its consumption in the fourth and highest quintiles was statistically significantly associated with a decreased prevalence of periodontal disease, showing a clear inverse dose-response relationship. After adjustment for cigarette smoking, toothbrushing frequency, region of residence, and body mass index, the inverse association was slightly attenuated but still remained statistically significant (adjusted OR = 0.65 [95% CI: 0.45–0.93] and 0.68 [95% CI: 0.47–0.97] for the fourth and highest quintiles, respectively; *P *for trend = 0.01). An independent inverse association was observed between tofu intake and the prevalence of periodontal disease: adjusted ORs were 0.59 (95% CI: 0.40–0.86) and 0.68 (95% CI: 0.47–0.98) in the fourth and highest quintiles, respectively (*P *for trend = 0.004). An inverse dose-response relationship between miso soup intake and the prevalence of periodontal disease was statistically significant but completely disappeared after multivariate adjustment; only miso soup consumption in the fourth quintile was independently related to a reduced prevalence of periodontal disease, however. No significant association was found between intake of tofu products, fermented soybeans, boiled soybeans, or miso and the prevalence of periodontal disease.

**Table 3:** Odds ratios (ORs) and 95% confidence intervals (CIs) for periodontal disease by quintiles of soy product intake in 3956 Japanese females

	Quintile of intake					
		
Variable	1	2	3	4	5	*P *for trend
Total soy product						
Intake, g/day^1^	13.1	26.2	37.4	53.6	86.8	
Prevalence	82/791 (10.4%)	66/791 (8.3%)	64/791 (8.1%)	52/791 (6.6%)	54/792 (6.8%)	
Crude OR (95% CI)	1.00	0.79 (0.56, 1.11)	0.76 (0.54, 1.07)	0.61 (0.42, 0.87)	0.63 (0.44, 0.90)	0.004
Adjusted OR (95% CI)^2^	1.00	0.80 (0.57, 1.13)	0.79 (0.56, 1.12)	0.65 (0.45, 0.93)	0.68 (0.47, 0.97)	0.01
Tofu						
Intake, g/day^1^	3.2	8.9	13.6	24.9	49.7	
Prevalence	77/791 (9.7%)	72/791 (9.1%)	72/791 (9.1%)	45/791 (5.7%)	52/792 (6.6%)	
Crude OR (95% CI)	1.00	0.93 (0.66, 1.30)	0.93 (0.66, 1.30)	0.56 (0.38, 0.82)	0.65 (0.45, 0.94)	0.002
Adjusted OR (95% CI)^2^	1.00	0.95 (0.67, 1.33)	0.96 (0.68, 1.34)	0.59 (0.40, 0.86)	0.68 (0.47, 0.98)	0.004
Tofu products						
Intake, g/day^1^	-0.6	0.6	1.5	3.4	6.7	
Prevalence	64/791 (8.1%)	54/791 (6.8%)	69/791 (8.7%)	67/791 (8.5%)	64/792 (8.1%)	
Crude OR (95% CI)	1.00	0.83 (0.57, 1.21)	1.09 (0.76, 1.55)	1.05 (0.74, 1.51)	1.00 (0.70, 1.43)	0.60
Adjusted OR (95% CI)^2^	1.00	0.83 (0.57, 1.21)	1.07 (0.75, 1.54)	1.05 (0.74, 1.51)	0.99 (0.69, 1.43)	0.60
Fermented soybeans						
Intake, g/day^1^	0	3.1	5.6	12.0	29.1	
Prevalence	65/791 (8.2%)	77/791 (9.7%)	70/791 (8.9%)	49/791 (6.2%)	57/792 (7.2%)	
Crude OR (95% CI)	1.00	1.21 (0.85, 1.71)	1.08 (0.76, 1.55)	0.74 (0.50, 1.08)	0.87 (0.60, 1.25)	0.07
Adjusted OR (95% CI)^†^	1.00	1.24 (0.87, 1.76)	1.14 (0.80, 1.63)	0.79 (0.54, 1.17)	0.94 (0.64, 1.36)	0.19
Boiled soybeans						
Intake, g/day^1^	-1.0	1.0	2.3	3.8	9.3	
Prevalence	68/791 (8.6%)	65/791 (8.2%)	60/791 (7.6%)	56/791 (7.1%)	69/792 (8.7%)	
Crude OR (95% CI)	1.00	0.95 (0.67, 1.36)	0.87 (0.61, 1.25)	0.81 (0.56, 1.17)	1.02 (0.71, 1.44)	0.77
Adjusted OR (95% CI)^2^	1.00	0.97 (0.68, 1.38)	0.88 (0.61, 1.26)	0.80 (0.55, 1.16)	1.04 (0.73, 1.48)	0.81
Miso						
Intake, g/day^1^	-0.6	0	0.4	0.8	6.2	
Prevalence	63/791 (8.0%)	63/791 (8.0%)	58/791 (7.3%)	66/791 (8.3%)	68/792 (8.6%)	
Crude OR (95% CI)	1.00	1.00 (0.70, 1.44)	0.91 (0.63, 1.33)	1.05 (0.73, 1.51)	1.09 (0.76, 1.55)	0.60
Adjusted OR (95% CI)^2^	1.00	1.00 (0.69, 1.44)	0.91 (0.63, 1.32)	1.04 (0.72, 1.49)	1.09 (0.76, 1.57)	0.61
Miso soup						
Intake, g/day^1^	0.2	1.9	4.3	6.9	11.5	
Prevalence	70/791 (8.9%)	78/791 (9.9%)	64/791 (8.1%)	43/791 (5.4%)	63/792 (8.0%)	
Crude OR (95% CI)	1.00	1.13 (0.80, 1.58)	0.91 (0.64, 1.29)	0.59 (0.40, 0.87)	0.89 (0.62, 1.27)	0.04
Adjusted OR (95% CI)^2^	1.00	1.14 (0.81, 1.61)	0.97 (0.68, 1.39)	0.63 (0.42, 0.93)	0.96 (0.67, 1.37)	0.12

^1^Values for intake are medians for adjusted energy intake by the residual method for each quintile.

^2^Adjusted for cigarette smoking (never and ever), toothbrushing frequency (1 or less, 2, and 3+ times/day), region of residence (Hokkaido and Tohoku, Kanto, Hokuriku and Tokai, Kinki, Chugoku and Shikoku, and Kyushu), and body mass index as a continuous variable.

Table 4 provides results in relation to consumption of soy protein and isoflavones. For dietary intake of isoflavones, daidzein, and genistein, the multivariate ORs for comparison of the fifth with the first quintile were of borderline significance, although the inverse linear trends were statistically significant (*P *for trend = 0.04, 0.05, and 0.04, respectively). There was no material association between soy protein intake and the prevalence of periodontal disease in the multivariate model.

**Table 4:** Odds ratios (ORs) and 95% confidence intervals (CIs) for periodontal disease by quintiles of soy protein and isoflavone intake in 3956 Japanese females

	Quintile of intake					
		
Variable	1	2	3	4	5	*P *for trend
Soy protein						
Intake, g/day^1^	1.3	2.7	3.8	5.5	9.0	
Prevalence	72/791 (9.1%)	75/791 (9.5%)	59/791 (7.5%)	57/791 (7.2%)	55/792 (6.9%)	
Crude OR (95% CI)	1.00	1.05 (0.75, 1.47)	0.81 (0.56, 1.15)	0.78 (0.54, 1.11)	0.75 (0.52, 1.07)	0.03
Adjusted OR (95% CI)^2^	1.00	1.08 (0.77, 1.53)	0.85 (0.59, 1.22)	0.83 (0.58, 1.20)	0.81 (0.56, 1.17)	0.10
Isoflavone						
Intake, mg/day^1^	7.3	15.3	21.5	31.9	52.7	
Prevalence	79/791 (10.0%)	68/791 (8.6%)	61/791 (7.7%)	56/791 (7.1%)	54/792 (6.8%)	
Crude OR (95% CI)	1.00	0.85 (0.60, 1.19)	0.75 (0.53, 1.07)	0.69 (0.48, 0.98)	0.66 (0.46, 0.94)	0.01
Adjusted OR (95% CI)^2^	1.00	0.86 (0.61, 1.21)	0.79 (0.55, 1.12)	0.73 (0.51, 1.04)	0.71 (0.49, 1.01)	0.04
Daidzein						
Intake, mg/day^1^	2.8	5.8	8.1	12.0	19.9	
Prevalence	77/791 (9.7%)	69/791 (8.7%)	62/791 (7.8%)	56/791 (7.1%)	54/792 (6.8%)	
Crude OR (95% CI)	1.00	0.89 (0.63, 1.25)	0.79 (0.55, 1.12)	0.71 (0.49, 1.01)	0.68 (0.47, 0.97)	0.01
Adjusted OR (95% CI)^2^	1.00	0.90 (0.64, 1.26)	0.83 (0.58, 1.18)	0.75 (0.52, 1.08)	0.73 (0.50, 1.04)	0.05
Genistein						
Intake, mg/day^1^	4.6	9.5	13.4	19.8	32.9	
Prevalence	78/791 (9.9%)	68/791 (8.6%)	62/791 (7.8%)	56/791 (7.1%)	54/792 (6.8%)	
Crude OR (95% CI)	1.00	0.86 (0.61, 1.21)	0.78 (0.55, 1.10)	0.70 (0.49, 0.99)	0.67 (0.46, 0.96)	0.01
Adjusted OR (95% CI)^2^	1.00	0.87 (0.62, 1.23)	0.81 (0.57, 1.16)	0.74 (0.51, 1.06)	0.72 (0.50, 1.03)	0.04

^1^Values for intake are medians adjusted for energy intake by the residual method for each quintile.

^2^Adjusted for cigarette smoking (never and ever), toothbrushing frequency (1 or less, 2, and 3+ times/day), region of residence (Hokkaido and Tohoku, Kanto, Hokuriku and Tokai, Kinki, Chugoku and Shikoku, and Kyushu), and body mass index as a continuous variable.

To examine whether the association of total soy product consumption with the prevalence of periodontal diseases could be attributed to isoflavone intake, we conducted further analyses in which we adjusted for isoflavone intake. Results of such analysis adjusting for isoflavone intake showed that the statistically significant inverse association between total soy product consumption and the prevalence of periodontal disease had completely disappeared: the adjusted ORs from the lowest to highest category of intake by quintiles were 1.0, 0.75 (95% CI: 0.45–1.27), 0.72 (95% CI: 0.38–1.37), 0.55 (95% CI: 0.26–1.18), and 0.57 (95% CI: 0.24–1.33), respectively.

## Discussion

This is the first epidemiological study to assess the relationship of soy and isoflavone intake and periodontal disease using a validated questionnaire with a broad range of categories of soy products. The present study found a statistically significant inverse association between consumption of total soy product, tofu, isoflavones, daidzein, and genistein and the prevalence of periodontal disease, whereas there was no measurable dose-response association between intake of tofu products such as deep-fried tofu and fried bean curd, fermented soybeans, boiled soybeans, miso, miso soup, or soy protein and the prevalence of periodontal disease. Our results showed that a high level of consumption of soy and isoflavone was associated with an approximately 30% decreased prevalence of periodontal disease. This finding appears to show moderate beneficial effects on periodontal disease. However, results of studies that assessed associations between soy or isoflavone intake and breast cancer and allergic rhinitis in Japan indicated a higher estimate of benefit than our results (approximately 50%) [7,12].

The Study of Women's Health Across the Nation demonstrated that median intake of daidzein and genistein by white subjects, African American subjects, and Japanese subjects in the US were 6.2 μg/d and 3.9 μg/d, 2.7 μg/d and 1.7 μg/d, and 4676 μg/d and 7151 μg/d, respectively [5]. The corresponding figures for the current study were 7640 μg/d and 12545 μg/d, respectively. The data available from a survey of dental disease in 1999 among Japanese aged 20 to 85+ years showed a prevalence value of 42.5% for periodontal disease with any pockets of 4 mm or greater [26]. On the other hand, 23.1% of US adults aged 30 to 90 years had periodontal disease with probing depth of 4 mm or greater according to the Third National Health and Nutrition Examination Survey [27]. Our findings point to a paradox in the prevalence of periodontal disease between Japan and Western countries that cannot be explained by dietary intake of soy and isoflavones. The difference in prevalence is likely to be a result of other environmental or genetic factors.

The underlying mechanisms for the observed inverse association between consumption of total soy products, tofu, isoflavones, daidzein, and genistein and the prevalence of periodontal disease are not known. Further control for isolfavone intake completely removed the inverse association between total soy product intake and the prevalence of periodontal disease. Therefore, the beneficial association may be ascribed to some extent to isoflavone intake or unmeasured constituents in relation to isoflavones. Because isoflavones are structurally analogous to human estrogens, they may combine with the estrogen receptor, albeit with lower affinity than estradiol, and stimulate estrogen activity [3]. Although some studies investigated a possible association between the use of oral contraceptives and periodontitis, evidence is not yet firmly established [18-20]. Normal circulating estrogen levels might be essential for periodontal protection [28]. Alternatively, dietary isoflavone intake might stimulate the host's immune system. A laboratory study demonstrated that interleukin-6, which is a central player in immune homeostasis, was regulated by isoflavones through estrogen receptors and gene expression mechanisms [29]. Another possible explanation is that healthy user bias may have confounded the observed relationship. Japanese who have a quite high intake of soy products are likely to follow the traditional Japanese diet or behaviors that may be preventive against periodontal disease. The observed beneficial effect of intake of soy products in this study might therefore be spurious.

The present study has a number of strengths and limitations. Our study population is inherently controlled for sex and age by restriction because the population consisted of female students aged 18 to 22 years. Our questionnaire included a wide range of soy products to estimate total intake of soy products and soy-related dietary components. Since 84.5% of the eligible subjects were included in this analysis, selection bias was likely to be negligible. We controlled for potential confounding factors, although information on socioeconomic status in the family, use of oral contraceptives, and oral health behavior other than tooth brushing, such as use of dental floss and professional tooth cleaning, were not available in this study. Several limitations of the study need to be considered, however. Outcome was determined by the subject's reporting on the question regarding a professional diagnosis of periodontal diseases. We did not conduct oral examinations of study participants. Nor was validity of the question assessed. A systematic review regarding validation of self-reported periodontal disease showed that knowledge of professional diagnosis of periodontal disease was more valid than responses by self-report to questions about disease awareness, symptoms, and treatment for periodontal disease [30]. That review indicated that one example of a good measure for self-reported periodontal disease was "Has any dentist/hygienist told you that you have deep pockets?", which had a sensitivity of 55% and a specificity of 90% against clinical pocket depth [30,31]. On the other hand, another study to validate self-reporting to an item, "Gums have bled recently," compared to the presence of any pockets with 4 mm or greater determined by clinical examination resulted in sensitivity of 25% and specificity of 88% [32]. Thus, self-reports of a professional diagnosis of periodontal disease would be likely to be an acceptable tool for epidemiological studies of periodontal health. The prevalence of periodontal disease in this study population (8.0%) appeared lower than that in a sample that consisted of Japanese women aged 20 to 24 years in a survey of dental disease in 1999 that showed that 13.1% had pockets of 4–6 mm [26]. Thus, it may be considered that the present study underestimated the presence of periodontal disease. Additionally, the inconsistency of diagnosis of periodontitis by dentists and the interpretation error by subjects may result in misclassification of periodontal disease. Random misclassification of participants' periodontal disease status across the 5 categories of exposures under study would have biased the estimates toward the null. The current study might not have substantial statistical power, although a significant inverse association was detected. The power of detection of the OR of 0.5 and 0.7 in the highest quintile compared with the lowest quintile was 97% and 57%, respectively (alpha error = 0.05, two-sided).

The estimations of isoflavone intake as well as soy consumption were derived from a self-administered semiquantitative dietary assessment questionnaire. Because the subject's dietary habits were not actually observed, we could only approximate consumption. However, participants with knowledge of professional diagnosis of periodontal disease might be aware of the ill effects of diet. The consequence would have been an underestimation of values in our results because of a nondifferential exposure misclassification. Because our study design was cross-sectional and both exposure and disease outcome were determined simultaneously, it was difficult to establish the time sequence of events; therefore associations between soy and isoflavone intake and periodontal disease do not necessarily indicate a causal relationship. The study subjects were women who were dietetics students aged 18 to 22 years, and they may differ from young women of the general population in terms of lifestyle characteristics, such as dietary habits. Thus, the present findings may be difficult to be generalized. Furthermore, we are uncertain whether the inverse association of soy and isoflavone intake with periodontal disease could also be found in Japanese men or in populations in other localities that consume low amounts of soy and isoflavone.

## Conclusion

This is the first epidemiological study to find an inverse dose-response relationship between soy and isoflavone intake and the prevalence of periodontal disease in young Japanese women. Further work is needed to confirm these provocative findings and to understand the mechanisms underlying this relation by using a more comprehensive database and studies with objective measures for severity of periodontal disease.

## Competing interests

The author(s) declare that they have no competing interests.

## Authors' contributions

SS, KM, HO, and YT contributed to the study concept and design and the acquisition of data. KT, SS and YM were responsible for the analysis and interpretation of data and the drafting of the manuscript. All authors participated in critically revising the manuscript and approved the final version of the manuscript.

## Pre-publication history

The pre-publication history for this paper can be accessed here:


